# Remote digital cognitive assessment reveals cognitive deficits related to hippocampal atrophy in autoimmune limbic encephalitis: a cross-sectional validation study

**DOI:** 10.1016/j.eclinm.2024.102437

**Published:** 2024-02-02

**Authors:** Kengo Shibata, Bahaaeddin Attaallah, Xin-You Tai, William Trender, Peter J. Hellyer, Adam Hampshire, Sarosh R. Irani, Sanjay G. Manohar, Masud Husain

**Affiliations:** a Nuffield Department of Clinical Neurosciences, University of Oxford, Oxford, OX3 9DU, UK; b Department of Brain Sciences, Imperial College London, London, W12 0NN, UK; c Department of Experimental Psychology, University of Oxford, Oxford, OX1 3PH, UK; d Centre for Neuroimaging Sciences, King's College London, London, SE5 8AF, UK

**Keywords:** Autoimmune limbic encephalitis, Hippocampus, Digital health, Remote cognitive testing, Online cognitive assessment

## Abstract

**Background:**

Autoimmune limbic encephalitis (ALE) is a neurological disease characterised by inflammation of the limbic regions of the brain, mediated by pathogenic autoantibodies. Because cognitive deficits persist following acute treatment of ALE, the accurate assessment of long-term cognitive outcomes is important for clinical assessments and trials. However, evaluating cognition is costly and an unmet need exists for validated digital methods.

**Methods:**

In this cross-sectional validation study, we investigated whether a remote digital platform could identify previously characterised cognitive impairments in patients with chronic ALE and whether digital metrics would correlate with standard neuropsychological assessment and hippocampal volume. Patients with ALE who had a chronic and stable presentation and received a clinical diagnosis of ALE were recruited for this study. The cognitive performance of 21 patients with ALE and 54 age-matched healthy controls — enrolled via the University of Oxford (UK) Cognitive Neurology Lab testing programme — was assessed with a battery of 12 cognitive tasks from the Cognitron online platform. The platform was optimised with National Institute for Health and Care Research (NIHR) support to be deliverable remotely to elderly and patient groups. The primary outcome measure was behavioural performance and corresponding neuroimaging and neuropsychological assessment metrics.

**Findings:**

Between February 15, 2021, and April 21, 2022, 21 patients with ALE (mean age 63.01 years, 14 males) and 54 healthy controls (mean age 65.56 years, 23 males) completed the digital cognitive assessment. Patients with ALE performed significantly worse in memory, visuospatial abilities, executive function, and language. No impairments in digit & spatial span, target detection (attention) and emotion discrimination were observed. The global score on the online cognitive tasks correlated significantly with the established Addenbrooke's Cognitive Examination III (ACE) pen-and-paper test. Deficits in visuospatial processing and language were identified in ALE compared to controls using remote digital testing but not using the ACE, highlighting higher sensitivity of computerised testing to residual cognitive impairment. Finally, the hippocampal volumes of patients with ALE and healthy controls correlated with online cognitive scores.

**Interpretation:**

These findings demonstrate that subtle cognitive deficits in patients with chronic ALE, who often show full recovery in measures of disability and dependence on daily activities, are detectable using a remote online platform, which also relates to hippocampal atrophy. Such methods may facilitate the characterisation of cognitive profiles in complex neurological diseases. Future longitudinal studies designed to assess the utility of such digital methods for further clinical characterisation are needed.

**Funding:**

The 10.13039/100010269Wellcome Trust, 10.13039/501100000265Medical Research Council, 10.13039/501100000272National Institute for Health Research, Rhodes Scholarship, and the Berrow Foundation Scholarship.

**Research in context**

**Evidence before this study**

A PubMed search for articles published between 2020 and 2023, using the terms ‘remote cognitive assessment’, returned 975 result, highlighting a considerable interest in the use of remote settings for cognitive testing. Remote administration of cognitive tests is becoming widely adopted in research settings for dementia. However, the cognitive domains investigated are limited. Furthermore, remote measures have scarcely been investigated together with neuroanatomical correlates, and no study has focused on remote cognitive measures in autoimmune limbic encephalitis (ALE) as a model cohort with chronic cognitive deficits.

**Added value of this study**

Using a validated online cognitive assessment tool, we assessed cognition in 21 patients with ALE. The online cognitive scores highly correlated with clinical neuropsychological scores and were also more sensitive to language and visuospatial deficits. Cognitive scores also correlated with the extent of hippocampal damage, the major focus of the pathology in ALE. This provides proof of principle for the utility of using such a test platform for remote assessment of cognitive function in long-term medical conditions.

**Implications of all the available evidence**

These results suggest that online remote cognitive assessment can support the assessment of cognitive decline in chronic and complex neurological conditions. Future work will be needed to assess the optimal task set to longitudinally identify cognitive deficits across neurological conditions for improved clinical decision-making.

## Introduction

Autoimmune limbic encephalitis (ALE) is an inflammatory disease that affects the structural integrity and functioning of the limbic system. Two frequently presented sub-types of ALE are characterised by autoantibodies against Leucine-rich glioma-inactivated 1 (LGI1) and Contactin-associated protein 2-Antibody (CASPR2).^1^ These antibodies are considered pathogenic and their transfer to rodents recapitulates cognitive aspects of the human conditions.^2^^,^^3^ Clinically, patients with ALE present with seizures, deficits in cognition and neuropsychiatric symptoms.^4^, ^5^, ^6^ However, the specific clinical and neuroimaging manifestations in these forms of ALE can vary widely across patients.^4^^,^^5^^,^^7^^,^^8^ Despite being a common feature, long-term cognitive deficits caused by ALE are still not fully characterised.^9^ Remote, multi-dimensional quantitation of patients’ cognitive profiles can help track disease activity and response to immunotherapies and are especially timely given the commencement of clinical trials in this field.

Immunotherapies improve outcomes in several ALE syndromes^4^^,^^7^^,^^10^^,^^11^ with most patients showing major improvements on the modified Rankin Scale (mRS) measure of disability and dependence on daily activities. Although cognition often improves on bedside testing (Montreal Cognitive Assessment (MoCA) and the Mini-Mental State Examination (MMSE)),^12^^,^^13^ residual and persistent deficits in memory and executive function are common^14^ even several years post-treatment.^4^^,^^9^^,^^12^^,^^15^, ^16^, ^17^, ^18^ Specifically, for LGI1-antibody encephalitis patients, deficits in episodic memory,^7^^,^^19^ working memory,^19^^,^^20^ language^12^ and fluency^12^ as well as fatigue^9^ are reported in post-acute phases of the disease. Despite this, long-term cognitive outcomes are not routinely assessed, likely due to the resources required to carry out in-person testing and the lack of validated tools.

Previous studies have found that surrogate digital measures may provide a rapid and cost-effective means of measuring long-term cognitive outcomes in a range of neurodegenerative disorders such as mild cognitive impairment (MCI),^21^ and Alzheimer's disease (AD).^22^ Recently, self-directed web-based computerised tasks have shown high validity with established clinical tests for MCI^23^ and sensitivity to changes in traumatic brain injuries (TBI)^24^ and early AD.^25^ Computerised testing also provides the opportunity to analyse trial-by-trial data to dissociate cognitive abilities from visuomotor response latencies.^26^ Such approaches highlight the use case of high-dimensional data that computerised testing can offer. Although further research is needed to validate the use of digital measures in clinical settings, these studies highlight the use of this technology to improve cognitive testing and overcome some of the limitations of in-person clinical assessments. Finally, the COVID-19 pandemic led to an increased demand for remote, contactless cognitive assessments. Investigations during the pandemic^27^, ^28^, ^29^ have demonstrated the feasibility of home digital testing across different age groups.

In this study, we used the Cognitron online cognitive assessment tool to investigate the cognitive profile of patients with chronic LGI1-and CASPR2-antibody encephalitis; two subtypes of ALE with similar neuropsychological profiles.^30^ Our objective was to identify domain-specific cognitive deficits using digital cognitive testing and correlate the results with the core behavioural and neuropsychiatric features of ALE. We compared performance on this digital testing platform with Addenbrooke's Cognitive Examination–III (ACE), a standard in-person clinical screening examination.^31^ In light of previous reports showing hippocampal atrophy in patients with ALE and associated cognitive deficits,^1^^,^^5^^,^^7^ we also assessed the relationship between hippocampal volume and cognitive outcomes. The evaluation of online cognitive assessment tools has the potential to facilitate the in-depth characterisation of long-term cognitive outcomes in ALE with important clinical utility.

## Methods

### Study design and participants

21 patients with chronic ALE (mean age = 63.01, SD = 8.14, 14 males) and 54 healthy controls (mean age = 65.56, SD = 7.31, 23 males) enrolled in the University of Oxford's Cognitive Neurology Lab testing programme. All patients with ALE had been clinically assessed and diagnosed by neurologists (MH, SGM and SI) at John Radcliffe Hospital in Oxford, UK. Cognitive assessment using Addenbrooke's Cognitive Examination III (ACE; Hsieh et al., 2013) was performed in 50/54 healthy controls and 20/21 patients with ALE. Magnetic resonance imaging was acquired in 47/54 healthy controls and 21/21 patients with ALE. Both were analysed retrospectively. All patients with ALE were clinically stable at the time of testing. Antibody positivity, treatments at the time of testing and the time from disease onset are reported in Supplementary Table S3. The ALE inclusion criterion was chronic with stable presentation, consistent with a clinical diagnosis of ALE. Participants provided written informed consent to participate in the study before taking part and were offered monetary compensation for their participation. This study was approved by the University of Oxford's Ethics Committee (18/SC/0048 & REC16/YH/0013).

### Behavioural paradigm

**Fig. 1:**
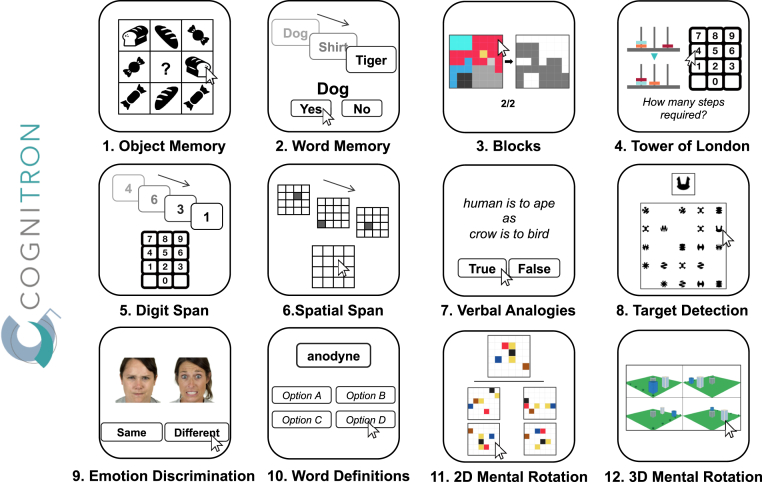
**Online Cognitron battery.** Twelve different cognitive tasks make up the Cognitron battery. Tasks were administered in the presented order, with object memory and word memory being tested at a delayed time point after all other tasks were completed. Feedback was provided after the completion of all tasks. The tasks were all run remotely in one sitting.

Patients with ALE and healthy controls were assessed using a modified version of the Cognitron protocol, an online battery of cognitive tasks that run via web browsers (available at: https://ompilot2.cognitron.co.uk/). The battery of tasks was programmed in HTML5 with JavaScript and hosted on the Amazon EC2 platform. The platform was optimised with National Institute for Health and Care Research (NIHR) support to be deliverable remotely to elderly and patient groups. This battery comprised 12 short cognitive tasks run in the same order for all participants, summarised below (Fig. 1). Participants accessed the tasks through a link on a web browser and were asked to run them on their own computers. Written instructions were provided at the start of the assessment and then at the start of each task. Feedback was provided after the completion of all tasks.

#### Cognitron tasks

##### Object memory

**Fig. 2:**
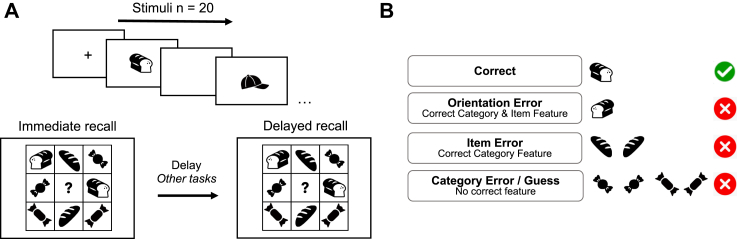
**Object memory task.** A) Twenty objects are presented sequentially. Immediately after the presentation, participants were required to identify an object presented in the initial sequence within an array of 8 objects of varying similarities, in random order. The same recognition task was administered at the end in delayed recall, on average 40 min later. B) Task performance was calculated using the accuracy of correct answers. The errors made were categorised into three different precision measures of recognition. Each has a varying number of correct features, in descending order.

Twenty black-and-white images of everyday objects were presented sequentially in random order. Presentation times were uniform at 2000 ms, with an inter-stimulus interval of 500 ms. Participants were tested on their memory of the objects immediately after the presentation in a multiple-choice array of 8 images (a 3 by 3 grid with an empty central box, Fig. 2A). One image was identical to one of the 20 objects presented prior and was the correct answer. The multiple-choice array also consisted of the identical but mirrored image, an object from the same semantic category (and its mirrored version) and 4 other images from a separate semantic category. Errors were categorised as either: 1) Spatial Error, 2) Item Error or 3) Category Error (guess), where Correct + Spatial Error + Item Error + Category Error = 1 (Fig. 2B). Delayed recall of the objects was tested a second time after all other cognitive tasks were completed. Participants were not informed that the memory of these objects would be probed at a delayed time point. The proportion of correct answers was used as the primary metric for object memory.

##### Word memory

Twelve English words were presented sequentially for 1000 ms with an interstimulus interval of 200 ms. The words were drawn from three categories: animals, clothes, and vegetables. Participants were asked to categorise a list of 24 words into old (seen in the earlier sequence) or new; 50% were targets (seen in the initial sequence of 12 words), and 50% were non-targets, of which half were lures with semantic similarity to the targets. Participants were also tested on the same set of words in a delayed recall after all other tasks in the battery, just after the delayed recall for the object memory Task. The delay time was comparable to that of the object memory delay. Correct choices and correct rejection of foil and lures were calculated. The number of correct answers was used as the primary performance metric.

##### Blocks

To assess spatial planning ability, participants were asked to match a configuration of tiles by removing shapes from a pre-configured layout of tiles. Importantly, the unsupported tiles fell down with gravity, changing the configuration. No time limit was enforced. The number of correct trials was used as the outcome variable.

##### Tower of London

A modified version of the Tower of London task was used to investigate spatial planning abilities. Participants were presented with two sets of three prongs with coloured beads on them. They were required to count the minimum number of moves it would take to change one set of three prongs to the other configuration and indicate the number on a number pad. Participants could not directly move the coloured beads and had to hold the number of moves in memory. There were 10 trials of varying difficulty, and the proportion correct was used as a measure of mental spatial planning.

##### Digit span

The digit span task required participants to hold numbers shown sequentially in memory. On each trial, participants were presented with a series of digits. At the end of the sequence, participants were required to recall the digits in the order they appeared by clicking on a number pad. Each successful trial led to an increase in the number of digits by one. The task was terminated when three consecutive errors were made on a given difficulty level. The maximum attained span was used as the memory measure, which ranged from 1 to 20.

##### Spatial span

Spatial short-term memory was tested using an adapted version of the Corsi Block Tapping Test. A sequence of locations was lit up in a 4 by 4 grid, and participants had to replicate the sequence. All trials began with two grids lighting up sequentially and were incremented by one on every successful recall. The task was terminated when three consecutive errors were made on a given difficulty level. The maximum sequence length for each participant was taken as a measure of spatial memory span, which ranged from 1 to 16.

##### Verbal analogies

Semantic (analogical) reasoning was tested by assessing the associations of two pairs of words. Participants were asked to indicate whether the association between words was true or false (e.g., ‘Human is to ape as crow is to bird’, true). Participants answered as many prompts as possible in 3 min. Every incorrect response led to a deduction of one point. Every correct point led to an increase of one point. The total score was used as a measure of word-based semantic reasoning.

##### Target detection

Spatio-visual attention was tested by presenting a target shape amongst a grid of dynamically changing shapes. The target shape remained on the left side of the screen for reference and remained unchanged for a given participant. In a 5 by 5 grid, new shapes were added every 1 s and others removed every 1 s. A target shape was added pseudo-randomly, at a frequency of 12 in every 20 new shapes. Participants were instructed to click on as many target shapes as possible. The task ended after 120 addition/removal cycles. The total number of target shapes clicked on was used as a measure of visuospatial target detection.

##### Emotion discrimination

The emotion discrimination task tested how well emotions are recognised by discriminating whether two facial emotions of different identities were the same or different. 50 trials were presented sequentially, and one point was awarded for every correct answer. No time limit was enforced in this task. The proportion of correct responses was used as the measure of emotion discrimination.

##### Word definitions

The word definition task tested semantic ability. Under each word, four definitions were provided. Each trial had to be completed within 20 s, and participants had to click on one of the four choices that most accurately defined the word. The proportion correct out of 21 trials was calculated.

##### 2D mental rotation

2D mental rotation was tested by presenting a 6 by 6 grid with a specific arrangement of coloured cells. This was the target grid presented next to four other grids. One of the four grids was an identical but rotated version of the target grid, which had to be selected. Participants had 3 min to complete as many trials as possible. The proportion of correct trials was used as a measure of 2D mental rotation.

##### 3D mental rotation

3D mental rotation was assessed by having participants find the rotated version of a 3D image of buildings that was the odd one out, where three configurations were identical when rotated. The task consisted of 12 trials, and the proportion of correct choices was used as a measure of 3D mental rotation.

### Magnetic resonance data acquisition

Structural Magnetic Resonance Images (MRI) were obtained from 20/21 patients with ALE and 47/54 healthy controls who were MRI-compatible. Participants were scanned at the John Radcliffe Hospital in Oxford using a 3T Siemens Verio scanner. T1-weighted structural images had 1 mm isotropic voxel resolution (MPRAGE, field of view: 208 × 256 × 256 matrix, TR/TE = 200/1.94 ms, flip angle = 8.62, iPAT = 2, pre-scan-normalise).

### Magnetic resonance data processing and analysis

Images were pre-processed with FMRIB Software Library (FSL) according to the UK Biobank analysis pipeline (https://git.fmrib.ox.ac.uk/falmagro/UK_biobank_pipeline_v_1).^32^ Hippocampal volumes were extracted from T1 anatomical images using FSL FIRST for segmentation and FSL SIENAX for volume extraction. Brain volumes were corrected for head size by the following equation^33^:

$${Volume}_{adjusted\;i\;}={Volume}_{raw\;i\;}-\;\beta \left({ICV}_{raw\;i\;}\;-\;{ICV}_{mean}\right)$$

where β is the slope of the line of regression between the ICV (Intracranial volume) and the adjusted volume. The time interval between in-person neuropsychological testing and the MRI scan was 61.03 days and did not differ between patients with ALE and controls (t (39.28) = −0.55, p = 0.58). The time between MRI and Cognitron task was 637.37 days and also did not differ between groups (t-test (t (21.64) = −0.30, p = 0.76). The time from scan and time from neuropsychological assessment did not affect the results when included as covariates.

### Statistical analysis

Statistical analysis was carried out on MATLAB R2021b and R Version 1.3.959. Equality of variance was tested using Levene's test and group comparisons were conducted using a two-tailed Welch's t-test, as the variance between groups was different. Linear regressions to assess correlations between variables were run by correcting for gender, age and years of education, and visualised using the *plotAdded* function on MATLAB. We reported p values for statistical significance (alpha level of 0.05), Cohen's d as a measure of effect size and the correlation coefficient r. No formal allowance for multiple testing was made. To provide a global score for cognition and reduce dimensions, a principal component analysis of the main outcome measure of all tasks was performed using the *pca* function in MATLAB. The first principal component was extracted as a global measure of cognition. The same dimensionality reduction analysis was carried out for the clustered domains of the Cognitron task set that shared cognitive processes. Network plots for inter-task correlations were assessed and visualised using the *network_plot* function in R, where a stronger correlation between tasks is represented by their spatial proximity and the colour of the lines connecting them. A logistic mixed effects model was performed using the *fitglm* function in MATLAB. The model was fit to the object memory task performance to describe the binary accuracy outcome of remembering the object as a function of Group (ALE or healthy control), Error Type (Spatial/Item/Category error) and Timepoint (Immediate/Delayed).) The analysis was run to assess the contribution of Group on accuracy, whilst taking into account different error types and the timepoint at which the data was collected while allowing for heteroscedasticity caused by unequal group sizes. We specified the random effects as the correlation between the Error Types within each subject. We accounted for the following within-subject variability, a) people who made more errors on one condition would make more errors on another condition, b) error slope (differences between the 3 error types) varying between people and c) people with a larger difference between error types making more errors overall. The following regression equation was used: *[Accuracy(correct/incorrect) ∼ 1* + *Timepoint + Group∗ErrorType* + *(1* + *ErrorType | Subject)].* No formal sample size calculation was carried out.

### Role of funding source

The funders had no role in the study design, data collection, analysis, decision to publish, or preparation of the manuscript.

## Results

### Demographics

Between February 15th 2021 and April 21st 2022, 21 patients with chronic ALE (mean age 63.01 years, 14 males) and 54 healthy controls (mean age 65.56 years, 23 males) completed the digital cognitive assessment. Healthy controls and patients with ALE did not differ in gender or in age. Patients with ALE had fewer years of education and as expected, a lower total ACE score (Table 1). Out of the 21 patients with ALE, 14 had LGI1-antibodies, and 5 had CASPR2-antibodies, 1 had both LGI1-antibodies and CASPR2-antibodies and 1 patient was seronegative. The seronegative patient showed clinical features similar to LGI1 ALE, with temporal lobe dysfunction. These include focal hippocampal atrophy, frequent seizures, loss of awareness, episodes of deja-vu, fatigue, confusion, and a good response to immunosuppressants, which they remained on at testing (Supplemental Table S3).

**Table 1:** Study demographics.

	Healthy control (n = 54)	ALE (n = 21)	Statistics
Gender			
Female (%)	31 (57.41)	7 (33.33)	χ^2^ (1, N = 75) = 3.51, p = 0.06
Age			
Mean years (SD)	65.56 (7.31)	63.19 (8.10)	t (33.35) = 1.16, p = 0.25, d = 0.31
Years of education			
Mean years (SD)	15.23 (2.81)	12.45 (2.44)	**t (40.76) = 4.09, p = 0.0002, d = 1.03**
ACE-III			
Mean score (SD)	97.46 (1.89)	92.45 (6.00)	**t (20.52) = 3.67, p = 0.0015, d = 1.42**

Healthy controls do not differ overall in age or gender to patients with autoimmune limbic encephalitis (ALE). The average years of education are higher in the healthy controls, although a high average of 12.45 years is reported for the patient group. Bold text indicates statistical significance (p < 0.05).

### Comparison of ALE and healthy controls

**Fig. 3:**
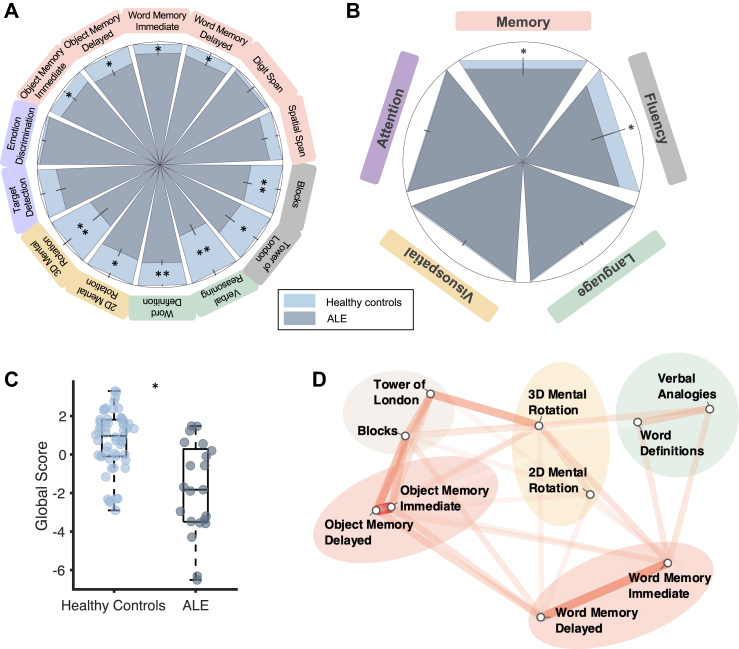
**Performance on Cognitron and pen and paper testing.** A) Group differences in performance on each Cognitron task were tested using Welch's t-test and visualised with a circular plot. Performance is scaled to the baseline performance of healthy controls (in light blue). Bars closer to the centre represent lower performance. ∗ = <0.05, ∗∗ = <0.01. Error bars are standard error of the mean. B) Group difference in Addenbrooke's Cognitive Examination (ACE) domains also using Welch's t-test revealed a difference in memory and fluency, but not in language, visuospatial abilities, or attention. Performance is scaled to the baseline performance of healthy controls. ∗ = <0.05. Error bars are standard error of the mean. C) A lower Global Score, calculated as the first principal component of all cognitive tasks combined, was found in autoimmune limbic encephalitis (ALE) patients compared to healthy controls. The boxplot represents the interquartile range, with the 50th percentile indicated by the median line. Vertical whiskers extend to values within 1.5 times the IQR from the quartiles. D) Network plot of task correlations (healthy controls only) reveals task clustering. Only metrics that significantly correlated with another task are included in the plot. A smaller distance and lower opacity of the connecting line indicate a stronger correlation.

The average group performance was assessed for each task. The average delay time between the immediate object memory task (run first) and the delayed object memory task (run last) was comparable between patients with ALE (42.45 min, SD: 7.76) and healthy controls (39.13 min, SD: 5.99), (t (29.76) = −1.77, p = 0.087). Healthy controls outperformed patients with ALE in 10 out of 14 tasks, (p < 0.05, d > 0.7) (Fig. 3A, See Supplemental Table S1 for statistics of group comparisons). In the neuropsychological ACE, healthy controls outperformed patients with ALE in the memory and fluency domains, but no difference was observed for language, visuospatial and attention domains (Fig. 3B, See Supplemental Table S2 for details of group comparisons). We did not find evidence showing that there is a significant difference in Cognitron task performance between LGI1 and CASPR2 ALE (Supplemental Figure S4).

### Clustering of online tasks yields five traditional cognitive domains

The first principal component from all cognitive measures of the computerised tasks was taken as a global cognition score. This component explained 32% of the variance in the dataset. The global score of cognition was higher in healthy controls (M = 0.71, SD = 1.53) compared to patients with ALE (M = −1.83, SD = 2.40), t (20.52) = 3.67, p = 0.0015, d = 1.42 (Fig. 3C). Analysis of correlations between individual task performances on healthy controls showed that the Cognitron tasks could be clustered into five domains: memory, planning, mental rotation, and language (Fig. 3D). Similar groupings were obtained with the patient group (Supplemental Figure S1). The strongest correlation observed in the Cognitron tasks was between the immediate and delayed version of the object and word memory tasks (object: r (73) = 0.77, p < 0.001; word r (73) = 0.59, p < 0.001).

### Cognitron performance correlates with ACE scores

**Fig. 4:**
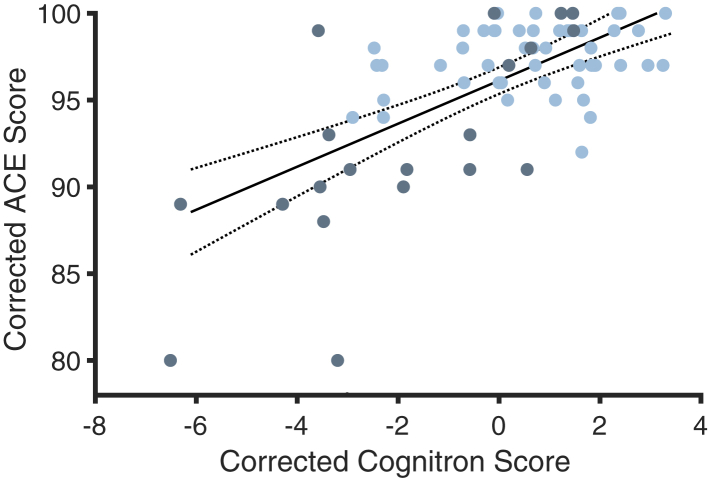
**Correlation between clinical ACE score and digital Cognitron score.** Linear regression between ACE score and Cognitron scores. The regression line is corrected for age, years of education and gender. The lighter blue dots indicate healthy controls, and the darker dots indicate patients with ALE.

We investigated the relationship between the global Cognitron score and ACE scores as a validation metric. Across all participants, the global Cognitron score was highly correlated with total ACE scores, whilst controlling for age, gender and years of education (r (49) = 0.46, p < 0.001, Fig. 4). This correlation remained significant when tested in patients with ALE separately (r (19) = 0.57, p = 0.02) but not in healthy controls (r (49) = 0.09, p = 0.09). This relationship is likely to be constrained by the limited variance of the ACE score in the healthy controls.

We took the first principal component of all computerised tasks in a single cognitive domain to compose a subdomain composite score (as grouped in the network plot, Fig. 3C). Each subdomain from the Cognitron battery was correlated with its corresponding ACE subdomain (memory, language, verbal fluency, visuospatial abilities, and attention). There was a significant correlation between Cognitron scores and the in-lab neuropsychological assessments in memory, attention and language domains (Supplemental Figure S2) showing that Cognitron domains mapped onto clinical neuropsychological test scores. Furthermore, while both the ACE and the Cognitron tasks captured deficits in memory and executive function (planning and fluency) in patients with ALE, only the Cognitron tasks detected deficits in visuospatial processing and language (Supplemental Table S2). Finally, neither ACE nor Cognitron subdomains showed a deficit in the attention subdomain for patients (Supplemental Table S2). The Cognitron tasks may therefore be sensitive to cognitive changes and show improved sensitivity to a decline in visuospatial function^34^ and language abilities^7^^,^^12^ compared to the standard neuropsychological test score.

### Memory deficits in ALE

#### Object memory task

**Fig. 5:**
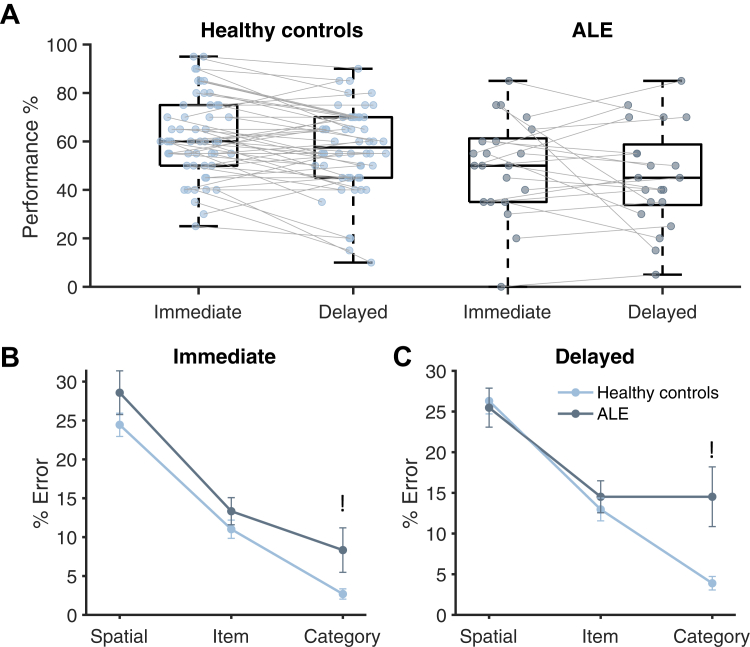
**Memory performance in patients with ALE compared to healthy controls.** A) Performance accuracy of the object memory score by group and delay. Both groups performed above chance for both conditions. The boxplot represents the interquartile range, with the 50th percentile indicated by the median line. Vertical whiskers extend to values within 1.5 times the IQR from the quartiles. B) % Error for each type of error made for immediate recall. Category errors were elevated in ALE compared to healthy controls. Error bars are standard error of the mean. C) % Error for each type of error made for Delayed recall. Category errors persist at delayed recall as well.

We examined memory tasks specifically in view of ALE being a disorder associated with medial temporal lobe and hippocampal lesions.^1^^,^^7^ The object memory task examined immediate and delayed recall of previously seen objects. Performance was significantly above chance level in both healthy controls and patients with ALE. Healthy controls outperformed the patients with ALE at both immediate and delayed time points (main effect of group F (1, 73) = 7.40, p = 0.008. and time point F (1, 73) = 2.36, p = 0.13) (Fig. 5A). No interaction between group and timepoint was observed (F (1, 73) = 2.47, p = 0.64).

To assess differences in performance between the two groups, we examined the types of errors made for all incorrect trials. Overall errors made on the task could be categorised into three types: 1) Spatial Error, 2) Item Error and 3) Category Error, in decreasing order of memory precision (Fig. 2B). While there was no main effect of the group on overall performance (β = 0.061, 95% CI = [−1.27, 0.25], t (8993) = 0.64, p = 0.52), patients with ALE made a significantly higher percentage of category errors compared to healthy controls (Group by category error interaction, β = 1.17, 95% CI = (0.48, 1.859), t (8993) = 3.34, p < 0.001. This highlights the behavioural difference in memory, which appears to be driven by an increase in low-resolution errors by patients with ALE who are getting the category of the objects wrong (Fig. 5B and C). As expected, a main effect of timepoint, where performance is lower at delay has been observed as well (β = 0.10, 95% CI = (0.0088, 0.20), t (8993) = 2.14, p = 0.032). This task, therefore, is able to identify a memory deficit in patients with chronic ALE that is not detected by the spatial and digit working memory span tasks which were not significant between the groups.

#### Word memory task

The word memory task had comparable immediate and delayed recall demands to the object memory task. In this task, patients with ALE generally performed worse than healthy controls, mainly in the delayed time point (main effect of group F (1, 73) = −2.53, p = 0.012 and time point F (1, 73) = −2.00, p = 0.046). At immediate recall, no significant differences were observed in the ability to reject lures in patients with ALE (M = 5.71, SD = 0.72) compared to healthy controls (M = 5.87, SD = 0.34), t (23.56) = 9.96, p = 0.35, d = 0.33), but healthy controls (M = 5.67, SD = 0.58) were more successful at rejecting foils compared to patients with ALE (M = 5.10, SD = 1.04), t (25.0) = 2.37, p = 0.026, d = 0.77).

### Hippocampal volume is associated with cognitron performance

Finally, we assessed the extent of hippocampal atrophy associated with ALE. Patients with ALE (M = 6881.98, SD = 1010.28) compared to healthy controls (M = 7498.65, SD = 575.67) had a significantly smaller bilateral hippocampal volume (t (25.99) = 2.61, p = 0.0015, d = 0.84). By comparison, there was no difference in the amygdala volume which is also a region of the medial temporal lobe, between patients with ALE (M = 2618.22, SD = 392.56) and healthy controls (M = 2552.92, SD = 362.05), (t (35.84) = −0.65, p = 0.52, d = 0.18). This indicates a specificity of atrophy to the hippocampus.

A linear regression between global Cognitron performance and total hippocampal volumes was performed across all study participants while controlling for age, gender, and years of education. This showed that a smaller hippocampal volume results in lower global cognitive scores (r (62) = 0.37, p < 0.001). When looking at the groups separately, the correlation was significant in the patients with ALE (r (15) = 0.67, p = 0.017) and not in healthy controls (r (42) = 0.08, p = 0.42). These correlations were driven by the patients with low hippocampal volume and expected low behavioural performance. All 4 subdomains of the Cognitron correlated with hippocampus volume (all p < 0.025) with the strongest relationship of the hippocampus to the language domain (r = 0.28) (Supplemental Figure S3).

### ALE treatments and performance on cognitron

An analysis of the effect of seizure medication, immunotherapy status (on/off) and prednisolone dosage (mg) at the time of testing on the global cognition score was conducted. Although the sample size is limited, no significant difference was observed in those on versus off seizure medication (t (1.42) = −0.05, p = 0.97, d = −0.03, Supplemental Figure S5–Panel A) or immunosuppression (t (8.30) = −0.10, p = 0.91, d = −0.05, Supplemental Figure S5–Panel B). Furthermore, no evidence supporting the hypothesis that dose of immunotherapy affects the global score of cognition was found r (10) = 0.01, p = 0.41, Supplemental Figure S5–Panel C).

## Discussion

In the present study, the cognitive profile of patients with ALE was compared to age- and gender-matched healthy controls using remotely administered online computerised tasks. People with ALE often present with cognitive deficits and focal atrophy of the medial temporal lobe.^1^^,^^5^^,^^7^^,^^35^, ^36^, ^37^ Therefore, it can serve as a model disease to study long-term cognitive impairments. Here, residual cognitive deficits in ALE (memory, language, visuospatial and planning) were found using the online platform and the outcome measures of this platform robustly correlated with standard cognitive screening. The online Cognitron platform also had higher sensitivity compared to the ACE to detect impairments in visuospatial abilities and language (Fig. 3A and B). Hippocampus-associated deficits in spatial and memory abilities were also evident, with an impairment in object memory, word memory and mental rotations tasks. Finally, hippocampal volume correlated with cognitive metrics from this online remote assessment, where a smaller volume was associated with worse deficits. The data supports the validity of remote digital testing as a clinical platform to assess long-term cognition in chronic ALE. This platform may also facilitate longitudinal monitoring which is not possible with traditional supervised assessment methods.

Patients with ALE, compared to healthy controls, performed worse across most domains including memory, visuospatial performance, and higher-order executive functions such as word definitions, verbal analogies, and planning. Such differences were reported with medium to large effect sizes (Cohen's d > 0.7). The deficits in 10 out of 14 tasks from the Cognitron battery are aligned with previous in-person studies reporting long-term cognitive deficits in ALE.^9^ No evidence for differences in cognition on the online platform was found between LGI1 and CASPR2 in any of the tasks. Although the sample size is limited, our present cohort suggests that cognitive performance is comparable in the ALE sub-types studied here.

The absence of deficits in patients with ALE in other domains (attention and emotion processing) may reflect the relative specificity of the antibody-mediated brain lesion in ALE. Target detection, a measure of attention, did not differ between groups, mirroring the pen-and-paper results (Attention domain in ACE). No difference in emotional discrimination was found between the two groups. Previous studies have reported impaired emotion processing at acute phases of the disease,^38^^,^^39^ and the evidence points to recovery after treatment. However, impaired emotion recognition^40^ and abnormalities in emotional regulatory processes^41^ have been reported in chronic ALE as well. Some residual impairments in emotional processes that are not reflected by an emotion discrimination task might still exist. A further investigation is necessary to confirm emotional processing in these patients.

We did not observe differences between ALE and healthy controls in working memory span tasks, although impairment in such tasks has been described^7^ (recovery of span tasks after 3 months has also been reported^42^). Importantly, longer-term memory deficits were observed in the object and word memory tasks. In the object memory task, we used everyday objects, available as a visual template which are easily integrated into cortical regions^43^^,^^44^ to facilitate encoding and retention of visual stimuli. A deficit in both the object memory and word memory tasks was found in immediate and delayed recall. In particular, an increase in guessing was observed in ALE during the object memory task. These results align with previous reports of memory deficits.^7^^,^^45^^,^^46^ Cognitron tasks also revealed visuospatial processing and language deficits in patients with ALE which have been reported in previous cohorts.^7^^,^^12^^,^^34^

Classically, the hippocampus is known to serve mnemonic and spatial functions.^47^ In ALE, autoimmune antibodies target limbic structures, particularly the hippocampus^1^^,^^5^^,^^35^, ^36^, ^37^ so it is unsurprising to observe worse spatial and memory performance in these patients. Evidence in temporal lobectomy studies have further highlighted the causal role of this brain region on such cognitive processes.^48^^,^^49^ As expected, the patients with ALE studied here had more hippocampal atrophy compared to healthy controls. Furthermore, hippocampal atrophy was related to the global score of cognition, including scores of memory and visuospatial capacity. This opens the door to probing hippocampal function using digital testing which will be helpful in other patient groups such as MCI and AD where the hippocampus is affected relatively early on in disease progression.

When comparing the Cognitron battery of tasks to standard neuropsychological tests, the cognitive composite score correlated with total ACE scores (Fig. 4). This was particularly pronounced in the patient group. Furthermore, the subdomains of the ACE and the Cognitron tasks were highly related. The expected deficits in language and visuospatial abilities were not captured by the in-person ACE, suggesting that the computerised tasks have greater sensitivity compared to pen-and-paper tests in these domains. Although standard neuropsychological tests remain the gold standard for cognitive testing, these results present evidence for the validity of remotely administered online cognitive assessments and supports its use as a valid alternative to traditional neuropsychological testing. There are several other major benefits to this mode of testing. Firstly, online remote assessment can allow for more widespread and convenient access to cognitive testing with high ecological validity. Secondly, remote online testing can facilitate the collection of larger sample sizes cheaply, as has been shown in Covid-19-related cognitive studies.^27^^,^^28^ Smaller study cohorts often face the concern of selection bias that may skew cognitive profiles. Digital assessments will allow recruitment from a wide participant pool and reduce the bias of socioeconomic status, cultural biases, and accessibility to testing sites. Thirdly, long-term follow-up can be performed, which is particularly relevant when assessing cognitive decline in neurodegenerative conditions^50^ Fourthly, immediate feedback can be provided which can be informative and motivating for participants and caretakers. Finally, although not leveraged in this study, detailed time courses of task performance can be used as a basis for computational modelling approaches that improve domain precision and sensitivity of cognitive ability estimates. These advantages make digital cognitive assessment an appealing research and screening tool for neurological disorders.

Crucially, this remote online tool is not intended to replace the current gold-standard diagnostic methods. Instead, cognitive testing may provide important additional information to guide diagnosis and treatment options in chronic patients. Here, we report a proof of concept that quantitative measures of cognition can be obtained remotely in a standardised manner. This may provide additional insight into clinical decisions on when to perform, potentially costly, imaging or in-person testing during the follow-up period of the disease and help decide diagnosis, treatment, and long-term management of these patients.

The focus on ALE was to evaluate digital testing in a real-world cohort with known hippocampus-specific disease processes. Here, Cognitron was tested on an autoimmune disorder affecting the temporal lobe. Validation in other patient groups, e.g., those with temporal lobe epilepsy, would be useful in further assessing the sensitivity of remote digital testing methods in people with a different cause of temporal lobe dysfunction. In addition, this study cohort includes patients with varied treatments and disease durations. Antibody status also varied (LGI1, CASPR2 and seronegative patients) which limits the specificity of these findings. Furthermore, the granularity of seizure frequency could not be reliably retrieved from patient records. Future studies targeted for the clinical characterisation of the effects of treatments, disease durations, disease severity and antibody differences using remote online testing, will provide important and novel insight into ALE. In the limited investigation here, we did not find evidence to support effects of treatments on remotely assessed cognition.

In the context of this study, we also note that healthy controls and patients with ALE were not matched for sex or years of education as a pre-existing participant cohort was used. While this limitation may introduce some variability into the reported findings, we have controlled for these differences by including sex and education level as covariates in the regression model. Future research with larger sample sizes could further explore the impact of sex, especially as LGI1 and CASPR2 ALE studies have also reported a male predominance.^4^

Overall, digital cognitive profiles may provide useful insight for treatment monitoring, prognostication, and improved diagnosis. We present a platform with the ability to collect self-administered cognitive data from a rare patient group with additional neuroimaging metrics. The global score of Cognitron measures correlated with in-person neuropsychological testing (ACE) and hippocampal volume, providing validity of online testing. Expected deficits, not identified by the ACE were identified using Cognitron. This approach may consistently measure behavioural changes across patients and generalise to other neurological diseases with cognitive deficits, providing a novel clinical platform for cognitive testing.

## Contributors

KS & MH designed the study. KS collected the behavioural data. BA collected and processed the MRI scans. KS and BA accessed and verified the underlying data. KS analysed the data with the help of BA, XT, & SGM. WT, PJH & AH designed and created the online cognitive testing platform. SRI, SGM, & MH provided access to patients with ALE. KS interpreted the results and drafted the manuscript with input from BA, XT, AH, SRI, SGM, and MH. All authors read and approved the final version of the manuscript.

## Data sharing statement

The datasets generated and analysed during the current study are available from the corresponding author upon reasonable request.

## Declaration of interests

AH and PJH are co-directors and owners of H2 Cognitive Designs Ltd. AH is the director and owner of Future Cognition Ltd, which supports online cognitive studies and develops custom cognitive assessment software respectively. SRI has received honoraria/research support from UCB, Immunovant, MedImmun, Roche, Janssen, Cerebral therapeutics, ADC therapeutics, Brain, CSL Behring, and ONO Pharma and receives licensed royalties on patent application WO/2010/046716 entitled ‘Neurological Autoimmune Disorders’ and has filed two other patents entitled “Diagnostic method and therapy” (WO2019211633 and US-2021-0071249-A1; PCT application WO202189788A1) and “Biomarkers” (PCT/GB2022/050614 and WO202189788A1). MH is a shareholder of Neu Health. No other author declares competing interests.
