# Supplementary material for: Remote digital cognitive assessment reveals cognitive deficits related to hippocampal atrophy in autoimmune limbic encephalitis: a cross-sectional validation study
Source: eClinicalMedicine. 2024 Feb 2;69:102437. doi: 10.1016/j.eclinm.2024.102437 (PMC10965407; doi:10.1016/j.eclinm.2024.102437)

**Title: Remote Digital Cognitive Assessment Reveals Cognitive Deficits Related to Hippocampal Atrophy in Autoimmune Limbic Encephalitis**

**Supplemental Table 1. Performance on Cognitron battery.** Differences in performance in Cognitron tasks between ALE patients and healthy controls compared using Welch’s T-test. Cohen's d is reported as a measure of effect size. The significant t-tests are highlighted in bold.


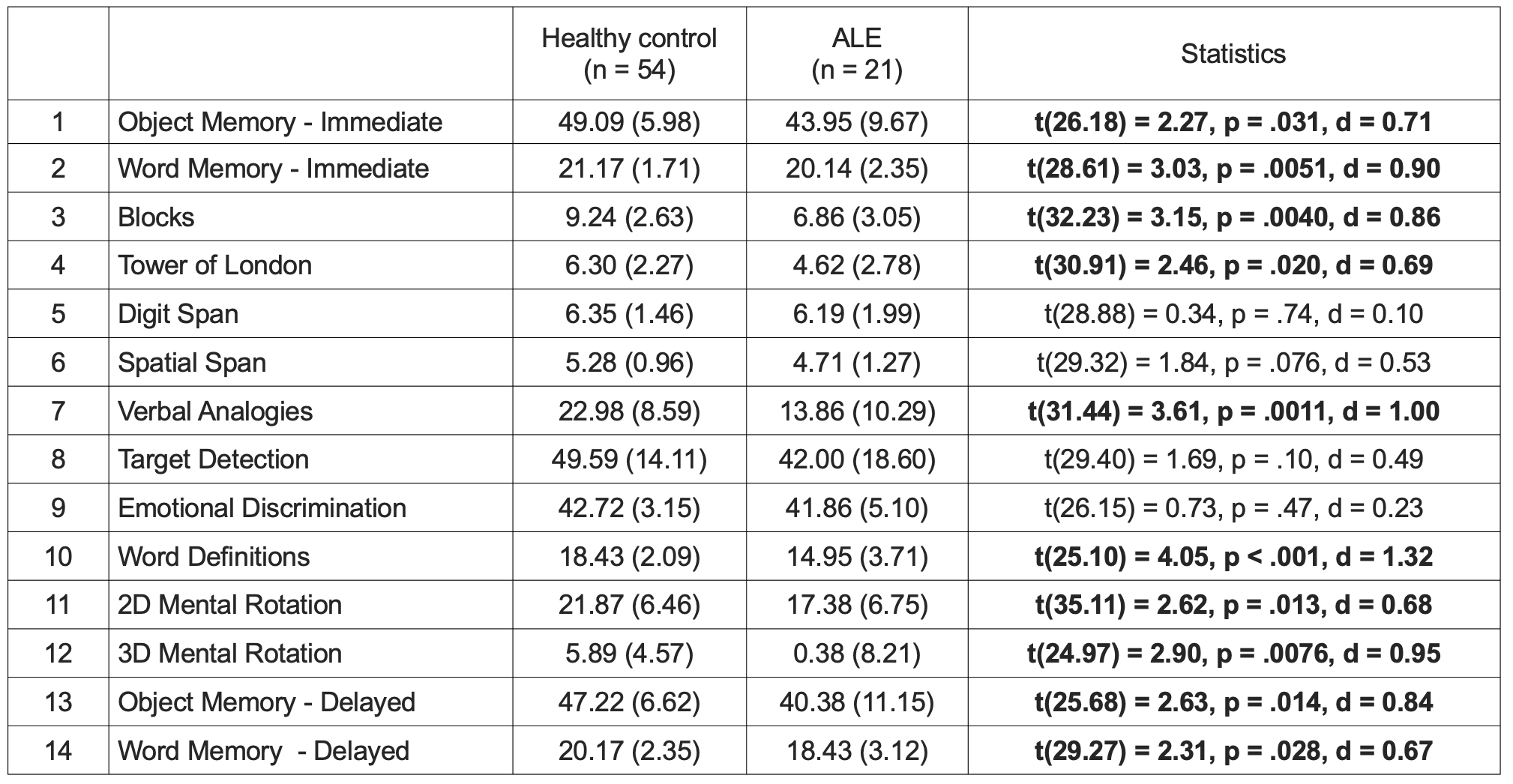


**Supplemental Table 2. Performance on Cognitron subdomains and ACE subdomains.** Differences in performance in each cognitive subdomain between ALE patients and healthy was compared using Welch’s T-test. Subdomains for the Cognitron tests were calculated by taking the first principal component of all tasks that make up the corresponding subdomain. Cohen's d is reported as a measure of effect size. The significant t-tests are highlighted in bold.

*
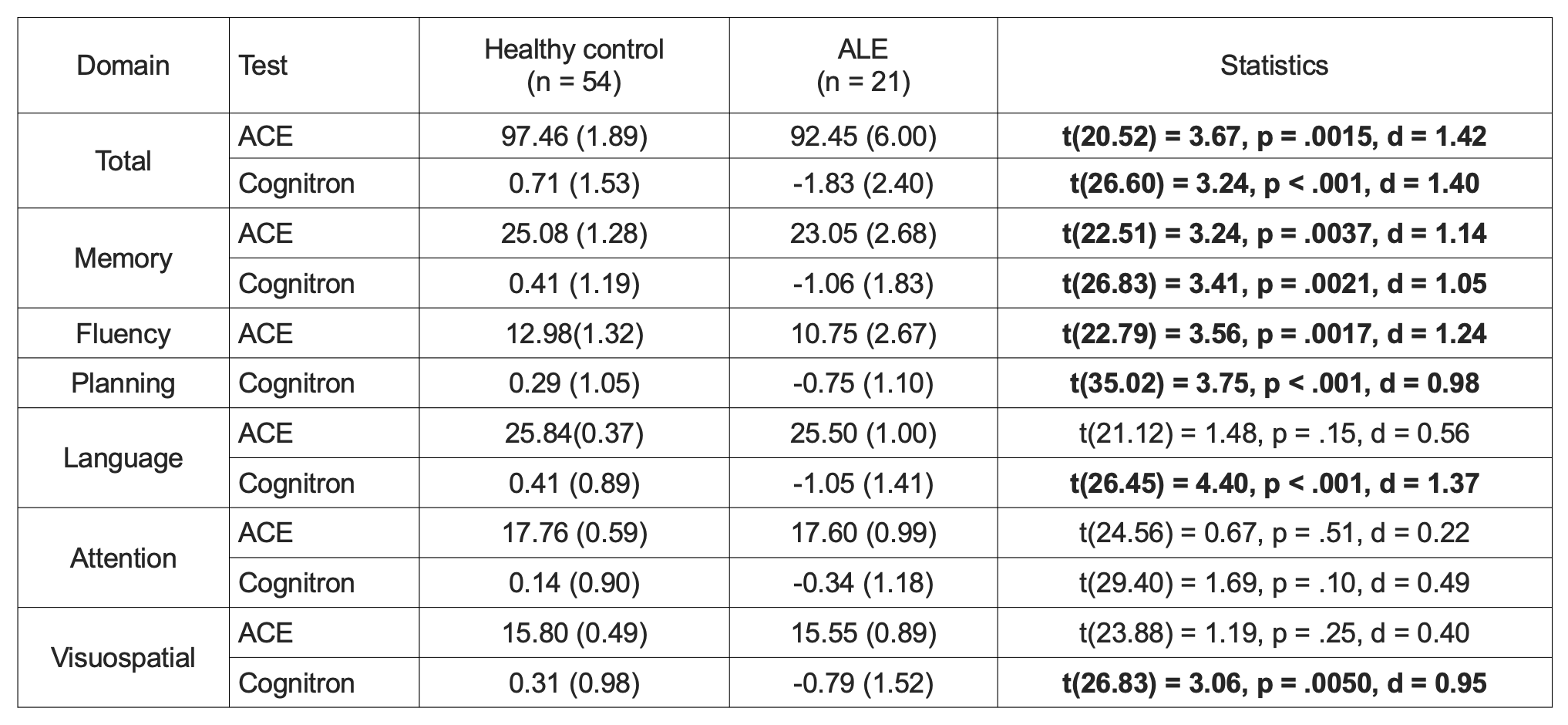
*

**Supplemental Table 3.** Treatment and disease time from onset at Cognitron testing. All patients were diagnosed with ALE and were in the chronic phase of the disease. Time from onset was calculated as the time of diagnosis to the time of testing.

| Patient No. | Antibody | Seizure medication | Long-term Immunotherapy (chronic phase) | Prednisolone  dose (mg) | Immunotherapy  at testing | Time from onset |
| --- | --- | --- | --- | --- | --- | --- |
| 1 | LGI1 | Carbamazepine | Prednisolone, Mycophenolate | n/a | Yes | 4 Yr 10 mo |
| 2 | LGI1 | Lacosamide, Carbemazepine | Prednisolone, Methotraxate | 20 | Yes | 5 Yr 1 mo |
| 3 | LGI1 | Levetiracetam | Prednisolone, Mycophenolate | 10 | Yes | 4 Yr 1 mo |
| 4 | LGI1 | Levetiracetam | Prednisolone | 0 | No | 3 Yr 6 mo |
| 5 | LGI1 | Lamotrigine | Prednisolone | n/a | Yes | 1 Yr 9 mo |
| 6 | Seronegative | Lamotrigine, Levetiracetam | Prednisolone | 10 | Yes | 2 Yr 12 mo |
| 7 | CASPR2 | Levetiracetam | Prednisolone | 40 | Yes | 0 Yr 7 mo |
| 8 | CASPR2 | Levetiracetam | none | 0 | No | 4 Yr 5 mo |
| 9 | LGI1 | Lamotrigine | none | 0 | No | 11 Yr 9 mo |
| 10 | LGI1 | Levetiracetam | Prednisolone | 7 | Yes | 5 Yr 1 mo |
| 11 | LGI1 | Lacosamide | Prednisolone, Mycophenolate | 0 | No | 5 Yr 0 mo |
| 12 | LGI1,  CASPR2 | Pregabalin | Prednisolone | 4 | Yes | 5 Yr 7 mo |
| 13 | LGI1 | Levetiracetam, Lamotrigine | Prednisolone | 5 | Yes | 5 Yr 4 mo |
| 14 | LGI1 | none | none | 0 | No | 5 Yr 1 mo |
| 15 | LGI1 | Levetiracetam | Prednisolone | 40 | Yes | 1 Yr 11 mo |
| 16 | LGI1 | none | Prednisolone | 40 | Yes | 1 Yr 8 mo |
| 17 | LGI1 | Levetiracetam | Prednisolone | 20 | Yes | 1 Yr 4 mo |
| 18 | LGI1 | Levetiracetam | Prednisolone | 25 | Yes | 0 Yr 9 mo |
| 19 | CASPR2 | Levetiracetam | Prednisolone | 30 | Yes | 3 Yr 5 mo |
| 20 | CASPR2 | Lamotrigine | n/a | 0 | No | 6 Yr 2 mo |
| 21 | CASPR2 | Lacosamide | n/a | 0 | No | 6 Yr 4 mo |

**Supplemental Figure 1. Cognitron task correlation.** Network plot of task correlations (healthy controls and ALE patients combined, n = 75). Only metrics that significantly correlated with another task are included in the plot. A smaller distance and lower opacity of the connecting line indicate a stronger correlation.


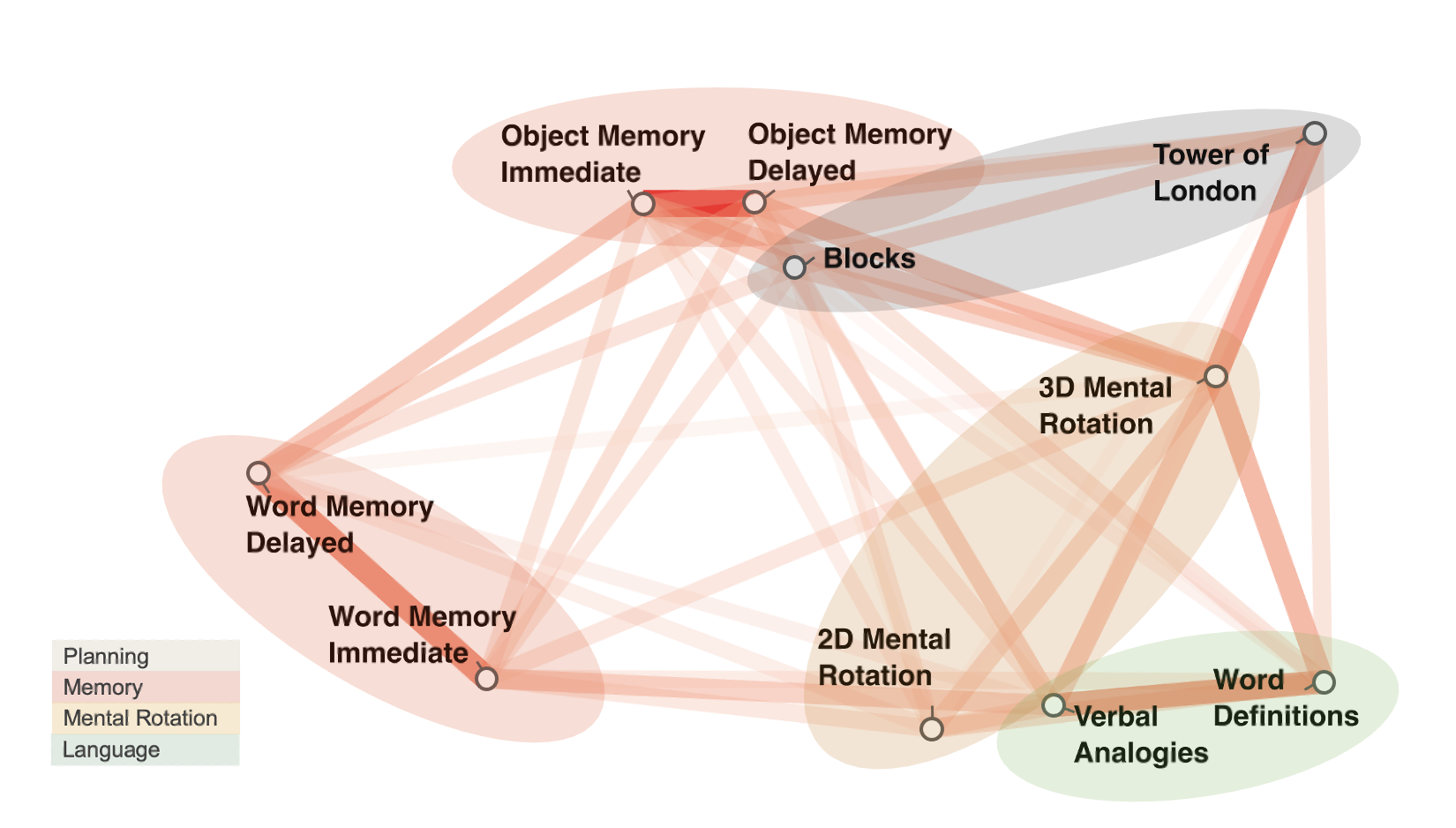


**Supplemental Figure 2. Correlation between ACE and Cognitron subdomains.** Correlation between clinical ACE score subdomains and digital Cognitron score subdomains.

**Supplemental Figure 3. Global Cognitron score and hippocampal volume.** Correlation between global Cognitron score and bilateral hippocampal volume.

**Supplemental Figure 4.** Comparison of LGI1 and CASPR2 patients’ performance on the Cognitron platform. Performance on each Cognitron task for 14 LGI1 and 5 CASPR2 patients is reported, with corresponding p values and Cohen’s d. One patient who was seronegative and another who tested positive for both LGI1 and CASPR2 antibody were not included in this analysis.

**Supplemental Figure 5.** Effect of seizure medication and immunosuppressants on Global Score of cognition from Cognitron. A) Global score of cognition did not differ between individual Off or On seizure medication. B) Global score of cognition did not differ between individual Off or On immunotherapy. C) The Global Score was correlated with the dose of immunotherapy at testing. No significant correlation was found.


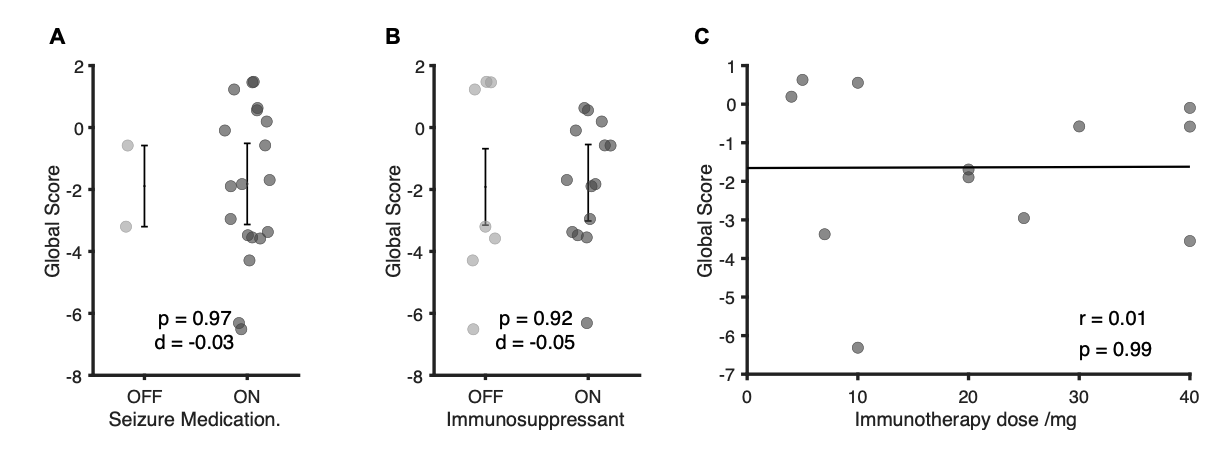

Supplement: Appendix [file mmc1.docx]
